# Treatment of large avulsion injury in perianal, sacral, and perineal regions by island flaps or skin graft combined with vacuum assisted closure

**DOI:** 10.1186/s12893-019-0529-1

**Published:** 2019-06-18

**Authors:** Fu Xing Hu, Xiao Xuan Hu, Xue Lin Yang, Xing Hai Han, Yong Bo Xu, Kun Li, Li Yan, Hai Bo Chu

**Affiliations:** 1 Department of burn and plastic surgery, the 89th Hospital of PLA, Weifang, 261021 China; 2 Department of Postgraduate, Ningxia Medical University, Yinchuan, 750004 China; 3 Department of general surgery, the 89th Hospital of PLA, Weifang, 261021 China

**Keywords:** Avulsion, Flap, Trauma, Wound closure techniques

## Abstract

**Background:**

Traumatic avulsion injuries to the anus, although uncommon, can result in serious complications and even death. Management of anal avulsion injuries remains controversial and challenging. This study aimed to investigate the clinical effects of treating large skin and subcutaneous tissue avulsion injuries in the perianal, sacral, and perineal regions with island flaps or skin graft combined with vacuum assisted closure.

**Methods:**

Island flaps or skin graft combined with vacuum assisted closure, diverting ileostomy, the rectum packed with double-lumen tubes around Vaseline gauze, negative pressure drainage with continuous distal washing, wounds with skin grafting as well as specialized treatment were performed.

**Results:**

The injuries healed in all patients. Six cases had incomplete perianal avulsion without wound infection. Wound infection was seen in four cases with annular perianal avulsion and was controlled, and the separated prowl lacuna was closed. The survival rate in 10 patients who underwent skin grafting was higher than 90%. No anal stenosis was observed after surgery, and ileostomy closure was performed at 3 months (six cases) and 6 months (four cases) after surgery, respectively.

**Conclusions:**

Covering a wound with an island flap or skin graft combined with vacuum assisted closure is successful in solving technical problems, protects the function of the anus and rapidly seals the wound at the same time.

## Background

Traumatic, large complete or incomplete soft tissue avulsion injuries in the perianal and perineal regions, due to their particular anatomic site (anterior urethra, posterior anus),are serious; they can result in complications, and their clinical management is challenging [1–4]. For surgeons and patients, the infection of surgical sitesis a challenging problem, and the incidence of infection ranges from 2 to 30% [5, 6].The treatment of traumatic wounds with the vacuum assisted closure (VAC) technique was first reported by Fleischmann in 1992, and was regarded as a milestone in later generations [7].Subsequently, the VAC technique has been widely used in trauma surgery, plastic surgery, general surgery, and burn surgery, and plays a key role in the prevention and control of wound infection and wound healing [8–10]. However, there are few literature reports on traumatic avulsion injuries in the perianal and perineal regions. In this study, 10 patients with large skin and subcutaneous tissue avulsion injuries in the perianal and perineal regions were treated with island flaps or skin graft combined with VAC, and their outcomes were determined.

## Clinical data and methods

### General information

Ten patients with large skin and subcutaneous tissue avulsion injuries in the perianal, sacral, and perineal regions following car accidents were enrolled from March 2010 to August 2016 in our hospital. There were eight men and two women with a mean age 35 years (range 28–45 years). The degree of damage was as follows: four cases had circumferential perianal avulsion, four cases had perianal avulsion on > 1/2 of the perimeter, and two cases had perianal avulsion on < 1/2 of the perimeter. These 10 cases also had avulsion injuries in the perineal region, six cases had skin and subcutaneous tissue defects which accounted for 8–25% of the body surface area, four cases had small intestine rupture, three cases had urethral disruption, two cases had penile and scrotal skin and subcutaneous tissue avulsion, two cases had vaginal tears, eight cases had pelvic fractures, and six cases had both lower limb fractures. Hemorrhagic shock was seen in eight cases and retroperitoneal hematoma in five cases. Five cases had secondary skin necrosis and four cases had necrotizing fasciitis. All patients had no anal canal and rectum damage (Table 1).

**Table 1:** Demographic characteristics of 10 patients with perianal avulsion injury

Patient No.	Gender	Age(Years)	Type of trauma	Assosiated Injuries	Peranal avulsion territory	Ostomy
1	M	28	MVA	PSSA, PF, UD, LLF	CPA	Yes
2	F	29	MVA	SIR, VT	CPA	Yes
3	M	39	MVA	SIR, PF	> 1/2 PAP	Yes
4	M	35	MVA	PSSA, UD	< 1/2 PAP	Yes
5	M	30	MVA	PF, LLF	> 1/2 PAP	Yes
6	M	37	MVA	SIR, PF, LLF	CPA	Yes
7	F	45	MVA	SIR, VT, PF	> 1/2 PAP	Yes
8	M	40	MVA	PF, LLF	> 1/2 PAP	Yes
9	M	28	MVA	PF, LLF	CPA	Yes
10	M	42	MVA	PF, LLF	< 1/2 PAP	Yes

Abbreviation: *M* male, *F* Female. *MVA* motor vehicle accident, *PSSA* Penile and scrotal skin avulsion, *PF* Pelvic fractures, *UD* Urethral disruption, *LLF* Lower limb fractures, *SIR* Small intestine rupture, *VT* Vaginal tears, *CPA* Circumferential perianal avulsion, *PAP* Perianal avulsion on perimeter

### Treatment

Correction of shock was performed quickly by supplementing the blood capacity(blood transfusion and fluid infusion),and preoperative preparation was quickly achieved. Early radical debridement was performed according to appropriate procedures under general anesthesia: necrotic skin and subcutaneous tissue were removed; adipose tissue was obtained in a prowl avulsion flap; residual normal skin was shaped in the perianal and genital regions. A local rotational flap was designed to maintain a distance of at least 2 cm between the normal skin and the anal margin.

**Fig. 1:**
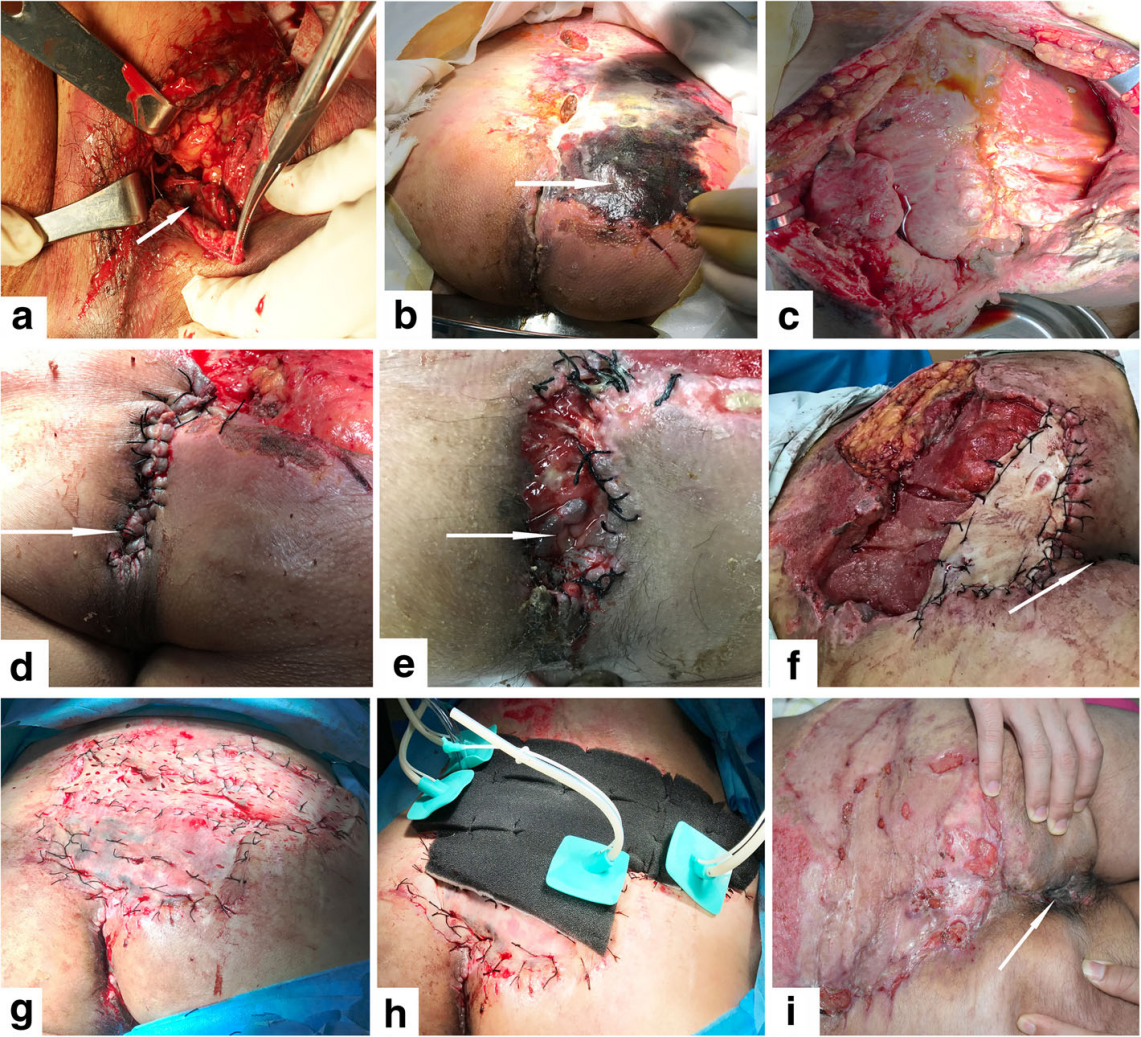
**a** Pictures of the treated wound in the patient with annular avulsion and anal retraction in lithotomy position (the arrow shows the edge of the anus). **b** 14 days after surgery, 12 × 8 cm skin necrosis, prowl separation area approximately 85 × 65 cm, the purulent exudate in the space, and the original fixed anus shortened (the arrow shows skin necrosis). **c** The necrotic tissue was resected, fully revealing the wound, and the abscess cavity was washed. **d** Resecting partial subcutaneous fat, interrupted suture of the flap and perianal skin, anal formation, vacuum assisted closure (VAC) coverage of the wound surface (the arrow shows the anus). **e**: 24 days after surgery, incision with silk thread, anal retracted, wound infection present (the arrow shows the anus). **f** A piece of free full thickness skin after excision, suture fixed near the anal side, the anus and wound isolated, the anus was reshaped, and the wound covered with VAC (the arrow shows the anus). **g** 34 days after surgery, the clean wound surface, fresh granulation, the prowl separation closed, incision healing in the shaped anus. The wound blocked by skin grafting. **h** The wound covered with VAC. **i** 48 days after surgery, wound and perianal skin healed (the arrow shows the anus)

Subcutaneous tissue and skin received layered interrupted sutures, the dead cavity was closed, and the anus and wounds were fully isolated. If the perianal skin and subcutaneous tissue injury were serious, and no local flap was available, then full-thickness or medium-thickness skin grafting was performed around the anus. Skin grafting width was 2–3 cm from the anal margin, and the length was in accordance with the wound around the anus. Suture fixation was achieved in order that VAC dressings could close the membrane, and the anus and wounds were isolated. Simultaneously, the avulsion wound was protected from intestinal bacteria present in the anus. Back and perineal avulsion injuries were fixed with a VAC dressing after debridement, with continuous negative pressure washing, and the VAC dressings were changed every 2 weeks after surgery. For patients with annular perianal avulsion, retraction of the anus and secondary subcutaneous infection can develop after surgery. During surgery, local rotational flap repair and discontinuous suspension plus U type sutures were used when possible, which reduced the degree of anal retraction as much as possible. At 2–4 weeks after surgery, granulation tissue formed in the avulsion wound, a split thickness skin graft was then carried out, and covered with VAC dressings (Fig. 1:a-i). A diverting ileostomy, the rectum was packed with double-lumen tubes around Vaseline gauze (for decompression and drainage as well as dilation of the anus), Redon-drainage tubes and vacuum bottles with continuous distal washing, wounds with skin grafting as well as specialized treatment were performed.

This study obtained approval from the Independent Ethics Committee of the 89th Hospital of PLA. Written informed consent was provided by the patients for their information. Patient records were anonymized and de-identified before analysis.

## Results

The injuries in all 10 patients healed. Of these 10 cases, six had incomplete perianal avulsion. During emergency surgery, debridement of the perianal skin, the rotational flap was stitched as well as the island flaps, the wound was covered with a VAC dressing, the secretion was drained via a multi-tube with negative pressure, and no wound infections occurred. Five patients had grinding contusions with secondary large areas of necrosis which healed after resection of the necrotic tissue and phase-II skin grafts. Four patients with annular perianal avulsion injuries were treated with anal formation plus island flaps, a VAC dressing was used to cover the wounds and continuous washing with multi-tube negative pressure drainage in the large separated cavity was carried out. Wound infection was controlled by rotational flaps plus anus formation and wound coverage with VAC dressings, the separated cavity was closed and the wounds healed after skin grafting. Ten patients with skin defects had two phase granulation wounds repaired with split thickness skin grafts, and the survival rate in 10 patients who underwent skin grafting was > 90%. Postoperative measurement of anal rectal pressure showed that static pressure, pushing pressure and the contraction duration time were normal. The rectal–anal inhibitory reflex was 130–140 ml (normal: 150–200 ml), and rectal maximum tolerance volume was 175–190 ml (normal: 200–600 ml).According to the pressure measurements, ileostomy closure was performed, and no anal stenosis or fecal incontinence in these patients was observed 6 months after surgery.

## Discussion

### Correcting shock and resolving anal contamination

Patients involved in car accidents have impact and contusion injuries, and shock in these patients can be fatal. Complex traumatic injuries canlead to misdiagnosis and delayed diagnosis. In the early treatment period, multi-disciplinary consultation and collaborative treatment are needed, and surgical decision-making must follow the principle of controlled injury [11, 12]. For anal skin avulsion, solving the problem of anal contamination is the key step in clinical treatment. For patients with circumferential perianal avulsion injury, anal formation was performed approximately 2 to 3 times plus island flaps, while VAC dressings covering the wound surface, continuous washing of multiple tube negative pressure drainage, wound infection control, and wound surface grafting to close the separated prowl cavity were also performed. Only by effectively solving the anal contamination problem can the surgeon create good conditions for wound healing. Therefore, the trauma of anal avulsion injury is mainly determined by the degree of perianal skin avulsion (circumferential avulsion or non-circumferential avulsion), and depends on the ability and clinical treatment experience of the decision-maker. However, to date, there are still no medical standards for treatment.

If the injury allows, the necrotic tissue should be removed, and the subcutaneous fascia tissue andmuscle tissue preserved with sufficient blood supply, which will create favorable conditions for granulation tissue growth and survival of the grafted skin. In the 10 patients with traumatic injuries included in this study, an ileostomy and placement of adual-chamber drainage tube were performed routinely, and continuous washing with negative pressure to minimize bacterial contamination from the anus was carried out [13, 14]. The rectum was packed with double-lumen tubes around Vaseline gauze and expansion of the anus was performed to prevent anal stenosis and for expected ileostomy closure. With regard to the indications for ileostomy closure, we actively recommend that the following conditions should be satisfied: (1) The anorectal static pressure, pushing pressure, duration of contraction, rectal-anal inhibitory reflex, and rectal maximum tolerated volume are almost normal; (2) A strong contraction of the anal sphincter is detected during the digital rectal examination; (3) For circumferential perianal avulsion, it is better to perform ileostomy closure at least 6 months after ileostomy. Following ileostomy closure, patients should continue to perform anal sphincter training and intermittent anal sphincter expansion, in order to restore the function of the anal sphincter and reduce anal stenosis as well as fecal incontinence.

### Preventing and controlling wound infection

For large skin and subcutaneous tissue avulsion injuries in the perianal, sacral, and perineal regions, it is very difficult for the surgeon to actively prevent and quickly control wound infection. During the wound healing process, inflammation (wound bacteria and disrupted cell migration), hyperplasia (revascularization, blood supply and nutrition), remodeling (fibroblasts entering the wound, secreting extracellular matrix and granulation tissue formation) processes take place [15]. The VAC technique can be beneficial in complex wounds by reducing the probability of multiple surgeries, relieving patient suffering and surgeon workload [16]. Animal experiments and clinical practice have confirmed that VAC with vacuum negative pressure (− 6.65 to − 16.63kpa), multi-tube drainage and continuous washing can prevent and control infection, promote granulation growth and accelerate wound healing [17, 18]. The VAC replacement time is inconsistent in the literature. Wang et al. [10] advocated that VAC should be replaced every 5–7 days. Liu et al. [19] believed that VAC should be replaced every 10–14 days. The VAC replacement time in the present study was consistent with that of Liu et al.

On the basis of application experience of VAC on skin avulsions of the extremities, we extended the application duration of VAC to treat skin avulsions on specific areas, and no skin necrosis occurred. We believe that this approach has the following advantages: (1) A relatively long time (7–14 days) facilitates the growth of granulation with good blood circulation and thus benefits the survival of skin grafts; (2). According to the size of the patient’s wound surface, continuous washing (with normal saline 250-1000 ml/day) and multi-tube negative pressure drainage, can avoid drainage tube blockage and minimize wound infection; (3) Due to the decreased frequency of VAC replacement, patients’ economic burden is reduced.

It is reported in the literature that VAC has the advantages of draining local fluid, reducing edema, decreasing the number of bacteria, increasing bacterial clearance rate, expanding small arteries, improving circulation and promoting granulation growth [20]. Four patients in the present study with perianal annular avulsion injuries complicated by infection underwent VAC twice, and wound infection was quickly controlled with wound granulation. Six patients with incomplete perianal avulsion injuries were treated with VAC dressings, and showed no infection and accelerated wound healing. The results of our study are consistent with those reported in the literature [10, 21]. We believe that VAC technology is the best choice for preventing and controlling infection, as it can minimize soft tissue necrosis and ensure the integrity of perineal skin in patients with perianal and perineal avulsion injuries.

### Choosing island flaps or skin graft

In patients with perianal and perineal avulsion injuries, integrity of the anal canal and sphincter function are normal under general circumstances. As rectal blood supply (rectal, middle and lower arteries) is rich, ischemic necrosis rarely occurs [4]. Traumatic large soft tissue avulsion injuries are classified as complete or incomplete, are often accompanied by extensive necrosis and severe contamination, which can damage the skin veins and capillaries as well as severely infect the blood supply and venous return, and can involve the muscles, nerves, bone and joints [22]. In patients with traumatic perianal avulsion injuries, the authors believe that rectal and visceral injuries should be excluded first [3].

Skin avulsion injury refers to the strong grasping traction caused by the external force rotation of the injured part, causing the skin to be separated between the subcutaneous tissue and the deep fascia of the muscle, forming a dead cavity-like change or a skin rupture tear. Avulsion injury can seriously damage the musculocutaneous artery or cutaneous artery that feeds the skin. If treatment of the traumatic conditionis not allowed or not timely, this often leads to progressive necrosis of skin and soft tissue, and eschar formation, which are the main clinical features of skin avulsion. The causes of skin necrosis are mostly direct damage, blunt shear and subcutaneous tissue prowl separation [23].

The generally accepted treatment strategy for avulsion injury is to treat the avulsed skin tissue with a full-thickness skin graft and reposition it with stabilization using either a local compression dressing or the VAC technique [24]. The skin surface of the avulsion injury often involves contusion injuries, and progressive necrosis of skin and soft tissue may occur. Surgeons treat wounds in an emergency situation, when the area of the avulsed skin is large, and the skin blood supply is compromised. For example, in one patient with annular perianal avulsion injuries, the blood supply in the injury was almost normal in the early stage (Fig. 1a). Five days after the injury, large areas of skin necrosis progressively appeared (Fig. 1b). The reason for this may be related to multiple factors, such as skin capillary damage and anal retraction leading to wound contamination. A large area with complete perianal and perineal avulsion injuries of the skin and soft tissue is a special situation; therefore, the early principles of debridement require consideration to treat the two issues, namely the wounds and preventing anal contamination, and involve multidisciplinary collaboration. In our experience, only local debridement sutures are needed in the early stage and contamination of wounds from the anus is controlled by ileostomy and Redon drainage tubes, and vacuum bottles with continuous distal washing of the rectum. When large areas of skin necrosis appear, the necrotic tissue should be removed. Free full-thickness skin grafting is used to establish an isolation zone between the anus and the wounds (Fig. 1f) and then free split-thickness or medium-thickness skin grafting plus the VAC technique are used, in order to increase the survival rate of the skin graft and control infection in the wounds. At the same time, the isolated region of skin grafting is also beneficial for sealing and adherence of the VAC dressing membrane in addition to the negative pressure drainage role of VAC (Fig. 1h).

In perianal and perineal incomplete avulsion injuries, secondary infection due to bacterial contamination from the anus should be prevented, and the authors often use rotational flaps plus anus formation and wound coverage with VAC dressings. In perianal and perineal complete avulsion injuries, anal retraction and subcutaneous prowl infection are prominent conditions. If a rotational flap plus anus formation is performed, the silk line has a cutting action on the skin and subcutaneous tissue due to the presence of tension, and inevitably causes tissue suture dehiscence. When the VAC vacuum effect disappears, infection is difficult to control. The authors selected island flaps or skin graft plus anus formation, followed by wound VAC coverage. This rotational flap (removing the subcutaneous adipose tissue) and anal formation can reduce local tension, and decrease the silk line cutting force on the skin and subcutaneous tissue. For full-thickness or medium-thickness skin grafting, it is easy to choose tough materials, which can close the wound, and achieve isolation of the anus and wound, thus controlling wound contamination.

As skin avulsion injury can cause blood circulation disorders in the basal part of the wound, the survival rate of full-thickness skin grafts was low, and multiple skin grafts were required. We used VAC to cover the wound for 2 weeks, which was beneficial for the formation of granulation tissue, and improved the survival rate of the skin grafts. At the same time, island flaps or skin graft can prevent bacterial contamination from the anus and adhesive VAC dressings can be used which will not be weakened on vacuum negative pressure. In the present study, the problem of anal retraction and wound contamination was successfully solved using island flaps or skin graft and the wound was sealed with skin grafts.

Traumatic avulsion injuries to the anus are difficult to treat due to fecal contamination and anatomical characteristics. In our study, an ileostomy was performed to prevent defecation and keep the perianal region clean. Island flaps or skin grafts were performed for avulsion injuries in the perianal, sacral, and perineal regions, and VAC therapy was then applied. However, the number of patients with different injury patterns was relatively limited. In addition, this was a retrospective study and the demographic data were not presented in detail.

## Conclusion

In the present study, the problem of anal retraction and wound contamination was successfully solved using island flaps or skin graft and the wound was sealed with skin grafts. Covering a wound with an island flap or skin graft combined with VAC was successful in solving technical problems, protects the function of the anus and rapidly seals the wound at the same time. This is an ideal treatment option for large skin and subcutaneous tissue avulsion injuries in the perianal, sacrum, and perineal regions.

## Data Availability

The datasets analyzed during the current study are available from the corresponding author on reasonable request.

## Abbreviations

CPA: Circumferential perianal avulsion
F: Female
LLF: Lower limb fractures
M: Male
MVA: Motor vehicle accident
PAP: Perianal avulsion on perimeter
PF: Pelvic fractures
PSSA: Penile and scrotal skin avulsion
SIR: Small intestine rupture
UD: Urethral disruption
VAC: Vacuum assisted closure
VT: Vaginal tears

## References

[1] Haas PA, Fox TA (1977). The importance of the perineal connective tissue in the surgical anatomy and function of the anus. Dis Colon Rectum.

[2] Jeganathan AN, Cannon JW, Bleier JIS (2018). Anal and perineal injuries. Clin Colon Rectal Surg.

[3] Ahern DP, Kelly ME, Courtney D, Rausa E, Winter DC (2017). The management of penetrating rectal and anal trauma: a systematic review. Injury..

[4] Terrosu G, Rossetto A, Kocjancic E, Rossitti P, Bresadola V (2011). Anal avulsion caused by abdominal crush injury. Tech Coloproctol.

[5] Sharma D, Rahaman H, Mandloi KC, Saxena A, Raina VK, Kapoor JP (2000). Anorectal avulsion: an unusual rectal injury. Dig Surg.

[6] Chen B, Hao F, Yang Y, Shang Q, Guo C (2017). Prophylactic vacuum sealing drainage (VSD) in the prevention of postoperative surgical site infections in pediatric patients with contaminated laparotomy incisions. Medicine (Baltimore).

[7] Fleischmann W, Strecker W, Bombelli M, Kinzl L (1993). Vacuum sealing as treatment of soft tissue damage in open fractures. Unfallchirurg.

[8] Kanakaris NK, Thanasas C, Keramaris N, Kontakis G, Granick MS, Giannoudis PV (2007). The efficacy of negative pressure wound therapy in the management of lower extremity trauma: review of clinical evidence. Injury..

[9] Vikatmaa P, Juutilainen V, Kuukasjarvi P (2008). Negative pressure wound therapy: a systematic review on effectiveness and safety. Eur J VascEndovascSurg.

[10] Wang J, Zhang H, Wang S (2015). Application of vacuum sealing drainage in the treatment of internal fixation instrument exposure after early postoperative infection. Minerva Chir.

[11] Cheong JY, Keshava A (2017). Management of colorectal trauma: a review. ANZ J Surg.

[12] Wu CW, Lin CH, Lin CH (2010). One-stage posttraumatic bladder reconstruction and soft-tissue coverage of the lower abdomen or perineum. Ann Plast Surg.

[13] Navsaria PH, Edu S, Nicol AJ (2007). Civilian extraperitoneal rectal gunshot wounds: surgical management made simpler. World J Surg.

[14] Velmahos GC, Gomez H, Falabella A, Demetriades D (2000). Operative management of civilian rectal gunshot wounds: simpler is better. World J Surg.

[15] Liu J, Hu F, Tang J, Tang S, Xia K, Wu S (2017). Homemade-device-induced negative pressure promotes wound healing more efficiently than VSD-induced positive pressure by regulating inflammation, proliferation and remodeling. Int J Mol Med.

[16] Langer V, Bhandari PS, Rajagopalan S, Mukherjee MK (2015). Negative pressure wound therapy as an adjunct in healing of chronic wounds. Int Wound J.

[17] Bulla A, Farace F, Uzel AP, Casoli V (2014). Negative pressure wound therapy and external fixation device: a simple way to seal the dressing. J Orthop Trauma.

[18] Liu Y, Hu DH, Dong ML, Wang YC, Liu JQ, Bai L (2011). Efficacy of vacuum sealing drainage in mice infected with Pseudomonas aeruginosa and its mechanism. Zhonghua Shao Shang Za Zhi.

[19] Liu X, Liang J, Zao J, Quan L, Jia X, Li M (2016). Vacuum sealing drainage treatment combined with antibiotic-impregnated bone cement for treatment of soft tissue defects and infection. Med Sci Monit.

[20] Ni J, Liu H, Liu X, Zhou L, Sun Y, Shi P (2015). Vacuum sealing drainage as treatment of severe buttocks and perianal infection: a case report and review of the literature (care-compliant). Medicine (Baltimore).

[21] Hu N, Wu XH, Liu R, Yang SH, Huang W, Jiang DM (2015). Novel application of vacuum sealing drainage with continuous irrigation of potassium permanganate for managing infective wounds of gas gangrene. J Huazhong Univ Sci Technolog Med Sci.

[22] Li RG, Ren GH, Tan XJ, Yu B, Hu JJ (2013). Free flap transplantation combined with skin grafting and vacuum sealing drainage for repair of circumferential or sub-circumferential soft-tissue wounds of the lower leg. Med Sci Monit.

[23] Hao S, Juan L, Xin W (2016). Treatment of traumatically cutaneous necrosis of buttocks using vacuum sealing drainage combined with ileostomy. Eur J Trauma Emerg Surg.

[24] Yan HD, Gao WY, Li ZJ, Wang CY, Liu S, Zhang F (2013). The management of degloving injury of lower extremities: technical refinement and classification. J Trauma Acute Care Surg.

